# Heterogeneous risk tolerance, in-groups, and epidemic waves

**DOI:** 10.3389/fams.2024.1360001

**Published:** 2024-04-05

**Authors:** Chénangnon Frédéric Tovissodé, Bert Baumgaertner

**Affiliations:** 1 Institute for Modeling Collaboration and Innovation, University of Idaho, Moscow, ID, United States; 2 Department of Politics and Philosophy, University of Idaho, Moscow, ID, United States

**Keywords:** standard of evidence, risk tolerance, in-group pressure, heterogeneity, behavior-disease dynamics

## Abstract

There is a growing interest in the joint modeling of the dynamics of disease and health-related beliefs and attitudes, but coupling mechanisms are yet to be understood. We introduce a model where risk information, which can be delayed, comes in two flavors, including historical risk derived from perceived incidence data and predicted risk information. Our model also includes an interpretation domain where the behavioral response to risk information is subject to in-group pressure. We then simulate how the strength of behavioral reaction impacts epidemic severity as measured by epidemic peak size, number of waves, and final size. Simulated behavioral response is not effective when the level of protection that prophylactic behavior provides is as small as 50% or lower. At a higher level of 75% or more, we see the emergence of multiple epidemic waves. In addition, simulations show that different behavioral response profiles can lead to various epidemic outcomes that are non-monotonic with the strength of reaction to risk information. We also modeled heterogeneity in the response profile of a population and find they can lead to less severe epidemic outcome in terms of peak size.

## 1 Introduction

There is a growing interest in informing public health by the joint modeling of the dynamics of disease and health-related beliefs, attitudes, and prophylactic behaviors [1-9]. A variety of factors and perspectives are likely involved in feedback loops between disease dynamics and prophylaxis. Many of these have been modeled, including, for instance, fear of infection mediated by messages from social circles or mass media [10-13], social influence [5, 14, 15], socioeconomic utility maximization [16-18], and evolutionary game theory [19-23]. Modeling such factors helps in understanding the determinants of the prophylactic responses of human populations to disease risk and is crucial for pre- or post-assessment of the effectiveness of causal interventions, including non-pharmaceutical interventions such as mask wearing, social distancing, and hand washing.

In this regard, it is well-known that risk tolerance varies in populations and largely explains the observed heterogeneity in responses to prophylactic behaviors such as mask-wearing [24-29]. To that end, Espinoza et al. [16] explored a system with adaptive behavioral responses where individuals privately adjust their contact rates by maximizing the utility of social interactions while minimizing infection risk. They found that an heterogeneous population of a risk-tolerant group with a risk-evader group can experience a more severe epidemic than an homogeneous population with no disparity in risk perception [16]. Similarly, heterogeneity in susceptibility resulting from heterogeneity in social activity can produce transient collective immunity as opposed to herd immunity, leading to multiple epidemic waves or plateau-like dynamics in heterogeneous populations [30, 31]. Accounting for heterogeneity in risk tolerance in behavior-disease models is thus important, not just academically but also for improving the soundness of contributions to public health policy design.

In that vein, it is important to recognize that the decision to wear a mask, for instance, is not a mere combination of risk tolerance and perceived prevalence of a disease. Mask-wearing is also informed by the aggregate behaviors of others, norms that can influence how people interpret the behavior of others in relation to disease prevalence, for example, suppose you visit a foreign town to attend a conference. When you arrive, you observe that there tends to be more people wearing masks than in your hometown. There are numerous inferences you can make from this evidence. Perhaps disease prevalence is higher. Maybe the risk tolerance tends to be lower. Maybe this town has implemented a policy but there is uneven compliance. Or further still, there might be in-groups that encourage mask-wearing. Suppose you notice a correlation between mask-wearers and people with conference badges. As a conference attendee yourself, you will likely feel some pressure toward wearing a mask, and if so, this will be because of how you have come to interpret the evidence of the mask-wearers.

Stated generally, it matters whether those behaving prophylactically are part of a larger in-group, because that impacts how people (both in and out of the group) interpret what the behavior is evidence of. We refer to this aggregate of in-group and behavior as the “interpretation domain.” There are few, if any, models that explore the dynamics of a system in which behavior contributes to and is a function of both perceived disease prevalence and an interpretation domain. For instance, evolutionary game theory-based models allow for individuals to engage or disengage in prophylactic behavior based on the relative importance of those already behaving prophylactically [20-22], but the decision process does not account for an interpretation domain. Interestingly, the social influence approach indirectly includes an interpretation domain: it assumes that constructive conversations between individuals with different risk tolerance levels can lead to opinion and attitude changes, and also includes an amplification mechanism by which individuals gain confidence in their health-related opinion after interaction with like-minded individuals [5]. However, social influence mechanisms cannot be directly observed [32], hence this approach lacks the perspective of directly fitting the models to observed epidemiological data and thus needs complementary methods to pair model predictions with observed data.

In this study, we propose a new behavior-disease compartmental model in which behavior contributes to and is also an explicit function of both perceived disease risk and an interpretation domain. As an example of context involving feedback loops that complicate the identification of the determinants of health-related behaviors at a population level, we consider the decision to wear a mask. For simplicity, the existing population level behavior-disease models largely consider Susceptible–Infected–Susceptible (SIS) or Susceptible–Infected–Removed (SIR) models for disease dynamic [14, 23, 33-35]. However, in a disease context with a significant proportion of asymptomatic infectives, the perceived risk of a person depends on incomplete information including the observed incidence (rather than the true incidence), and estimates of the disease prevalence publicized in media favored in the interpretation domain. Behavioral changes thus depend on the composition of the infective class, the probability to detect infectives, and testing effort, and this advises against the use of the SIR model. Furthermore, in some epidemic contexts, disease-related information, including both historical incidence and predicted trends, is discussed on a daily basis on mass media, possibly leading to risk information overload [36]. In such situations, for instance, during the COVID-19 pandemic, the decision to wear a mask may be affected by predicted trends in addition to historical risk.

Considering an hypothetical disease with COVID-19-like epidemiology, we use an extended Susceptible–Exposed–Infected–Removed (SEIR) model with differentiated infective states (asymptomatic, symptomatic, and detected infectious). Risk information from this disease dynamic comes in two flavors, namely the current disease prevalence and trends in number of new detected infectives. It flows into the behavior dynamic model with a time delay. Susceptible individuals consider this risk information along with how many individuals in their social group are already engaging to decide to (not) adopt a prophylactic behavior, which provides a certain level of protection against infection. Changes in prophylactic behaviors then flows back into the disease dynamic model through variations of the effective contact rate between infectives and the susceptible groups. The resulting model is a system of neutral delay differential equations [37], where changes in the disease state variables depend not only on the current states but also on the history of the system.

The behavior dynamic part of our model mimics a generalized logistic growth process [38] with explicit formulae (given observable disease risk information). This is an advantage over competing approaches such as the evolutionary game theory-based model [21] since, by carrying surveys before or during the early phase of an epidemic outbreak, one can obtain estimates of model parameters and derive predictions of disease-behavior co-evolution under various scenarios of interest to public health, and further iterate feedbacks between model and observable data. We do not, however, explore such an advantage here. Instead, we consider a variety of “response-profiles” inspired by work in behavioral economics and related fields [39-41]. For example, one kind of profile focuses on the current disease incidence rate. Another kind considers trends in an effort to “predict” incidence rates, thereby opening the possibility of adopting prophylactic behavior earlier when the trend moves upwards, but also giving up such behavior when the rate of change decreases. Such profiles can be further manipulated with increased (or decreased) levels of risk-aversion, as well as increased (or decreased) levels of in-group pressures (i.e., how much attention is given to pro-prophylactic behavior vs. non-prophylactic behavior in the interpretation domain).

Our purpose is to explore how differences in such response profiles affect disease dynamic. In particular, we consider epidemic severity in terms of epidemic peak time and size, time to curb the epidemic (effective reproductive number below one), final epidemic size, and the possibility of multiple epidemic waves. Under a social influence-based model, Tyson et al. [14] found that populations more responsive to risk information can experience more severe epidemics in terms of final size and undergo multiple epidemic waves, although the epidemic peak sizes will be smaller. Here, we aim to test these results: (*i*) when the prophylactic response to risk information involves in-group pressure (which is similar to the social influence mechanism, but can account for an interpretation domain) under various response-profiles (i.e., populations responding to qualitatively different risk information); and (*ii*) when the population is heterogeneous in terms of response-profile.

The remainder of the study is organized as follows. Section 2 describes the components of the new behavior-disease compartmental model in terms of equations, motivations, and interpretations, and derives summary measures considered to assess and compare epidemic severity and overall disease dynamic. Section 3 explores various aspects of epidemics that can be generated by the proposed differential system, considering populations homogeneous or heterogeneous with respect to in-group behavior and response to historical or predicted risk information. Section 4 then discusses our findings and the limitations of the proposed model, and provides some concluding remarks.

## 2 Methods

This section introduces our general theoretical framework based on an SEIR model. It then describes risk information components, as well as how the proportion of prophylactic individuals in a sub-population and the effective contact rate depend on this information and in-group pressure. Finally, the section presents the behavior-disease dynamics model and related summary measures to quantify and compare epidemic severity.

### 2.1 Theoretical framework

It is well-known that prophylactic behavior affects disease transmissions by reducing rates of effective contacts between infectious and susceptible individuals in the target population (direct physical or indirect through shared media in which pathogens can survive) [23, 42]. In our modeling framework, change in disease dynamic affects back prophylactic behavior through observable disease prevalence (which depend, for instance, on the existence and effectiveness of a disease surveillance mechanism and testing capacity to detect infectious individuals), but disease related information is processed by a system of health beliefs and related attitudes. In other words, the feedback from disease dynamic to prophylactic behavior is mediated by health beliefs and normative attitudes in the individual’s interpretation domain which determines risk tolerance. However, engagement in prophylactic behavior is also determined by how many individuals are already behaving prophylactically, and a social group can pay more attention to and mimick those behaving prophylactically than those behaving non-prophylactically, or vice-versa.

To describe disease dynamics, we consider an extended SEIR compartmental model framework [43-45] with differentiated infective states. The population size N is given at time t by

(1a) $$N(t)=S(t)+E(t)+I_{a}(t)+I_{s}(t)+I_{d}(t)+R(t)$$

where S, E, $I_{a}$, $I_{s}$, $I_{d}$, and R are susceptible, exposed, asymptomatic infectious, symptomatic infectious, detected (tested positive and reported), and removed individuals, respectively. The disease-dependent compartments E, $I_{a}$, $I_{s}$, $I_{d}$, and R are considered homogeneous whereas the class of susceptible individuals (S) is further differentiated into two groups based on risk tolerance:

(1b) $$S(t)=S_{-1}(t)+S_{1}(t)$$

where $S_{i}$ denotes susceptibles with standard of risk i∈𝒜, 𝒜={−1,1}. For instance, in a population which only responds to current risk, $S_{-1}$ may represent individuals with low standard of evidence (the most responsive to disease prevalence), while $S_{1}$ corresponds to individuals with high standard of evidence (least responsive to disease prevalence). However, difference in standard of risk may be qualitative rather than quantitative: $S_{-1}$ may represent individuals responsive to only historical risk while $S_{1}$ corresponds to individuals sensible to both historical and predicted risk.

### 2.2 Disease risk information aggregate

The primary source of disease-related information in an epidemic context is the timely number of new positive cases, denoted C. The number of individuals in the class $I_{d}$ of detected infectious individuals (see Equation 1a) is related to C by

(2) $${\dot{I}}_{d}(t)=C(t)-\rho_{d}I_{d}(t)$$

where the dot notation indicates the first derivative with respect to time (i.e., ${\dot{I}}_{d}(t)=dI_{d}(t)∕dt$), $\rho_{d}$ is the removal rate of individuals from $I_{d}$, with non-negative initial condition $I_{d}(0)=I_{d0}$. Solving the differential Equation (2) for $I_{d}$ gives

(3) $$I_{d}(t)=\text{exp}\;(-\rho_{d}t)\left[I_{d0}+\int_{0}^{t}C(u)\;\text{exp}\;(\rho_{d}u)\;du\right].$$

We consider the perceived disease prevalence, denoted P, and the relative rate of change of new positive cases, denoted Q, as the basic pieces of information on which susceptible individuals will decide to be prophylactic. From Equation (3), the perceived disease prevalence is given at time t by

(4a) P(t)={Id(t−τ)N(t−τ)ift≥τ,0otherwise}

where the constant τ>0 represents a time delay in the acquisition of information on detected and reported infectious. The relative rate of change Q is the quotient of the rate of change (increase or decrease) of the number of new detected cases C to the number of new detected cases, delayed by τ time units:

(4b) Q(t)={C.(t−τ)C(t−τ)ifC(t−τ)>0,0otherwise}.

Note that Q(t)=1 means that the timely number of new positive cases is doubling per unit time, whereas Q(t)=−1∕2 means that the timely number of new positive cases is halving per unit time.

We assume that based on the information pieces P and Q, each group of susceptible $S_{i}$ makes up an information aggregate, denoted $\eta_{i}$, and which satisfies $\eta_{i}\;=\;0$ before disease outbreak (t<τ, P=Q=0). This information aggregate is defined as a quadratic function of P and Q:

(4c) $$\eta_{i}=a_{i}P+b_{i}P^{2}+c_{i}PQ+d_{i}Q^{2}+e_{i}Q$$

where $a_{i}$, $b_{i}$, $c_{i}$, $d_{i}$, and $e_{i}$ are non-negative real coefficients expressing the weights of linear, quadratic, and interaction components of the prevalence P and the rate of change Q. It is worthwhile noticing that Equation (4c) is intended as an approximate summary of the available information that drives the decision to wear a mask. The quadratic form is indeed used as an approximation to the actual, likely non-linear mechanism by which the susceptible group $S_{i}$ processes disease-related information. The non-negative signs imposed on the coefficients in $\eta_{i}$ ($a_{i}$, $b_{i}$, $c_{i}$, $d_{i}$ and $e_{i}$) ensure that when the timely number of new positive cases is non-decreasing (Q≥0), $\eta_{i}$ is not only non-negative for any perceived disease prevalence P but also non-decreasing in P.

Equation (4c) describes how disease risk information can be differently interpreted by different tolerance groups within the same population. For instance, a group of individuals may focus on the disease incidence rate while ignoring predictions of future disease risk. For such individuals, one or both parameters $a_{i}$ and $b_{i}$ in the information aggregate (Equation 4c) will be positive but parameters $c_{i}$, $d_{i}$, and $e_{i}$ will be zero. Another group may pay attention to trend in disease incidence, opening the possibility of adopting prophylactic behavior earlier when the trend moves upwards, and also giving up such behavior when incidence decreases. Such a group will have at least one of the parameters $c_{i}$, $d_{i}$, and $e_{i}$ being greater than zero. Between these two extreme situations, there are many possibilities with various combinations of small vs. large values of parameters $a_{i}$ and $b_{i}$ to reflect how much attention is paid to current disease incidence by a tolerance group, and parameters $c_{i}$, $d_{i}$, and $e_{i}$ related to the relative importance of trends to the group.

### 2.3 Prophylactic behavior dynamic

For a susceptible class with standard of evidence i, we consider for simplicity two levels of prophylactic behavior: high prophylactic behavior (i.e., individuals properly wearing mask where and when this is recommended) vs. low prophylactic behavior. The overall prophylactic behavior in the class $S_{i}$ can thus be summarized by the prophylactic proportion $m_{i}\in [0,1]$, i.e., the proportion of mask-wearers. We assume that change in the prophylactic proportion $m_{i}$ is proportional to change in the information aggregate $\eta_{i}$ and to the proportion of $S_{i}$ individuals already wearing masks:

(5) $$\frac{\partial m_{i}}{\partial t}=\left[m_{i}\frac{1-m_{i}^{\alpha_{i}}}{\alpha_{i}}\right]\frac{\partial \eta_{i}}{\partial t}$$

where $\alpha_{i}$ is a positive real which determines the nature and strength of in-group behavior, and we have taken the proportionality constant to be one to ensure that the coefficients of the linear components of $\eta_{i}$ ($a_{i}$ and $e_{i}$) in the model are identifiable from observed data. When $\alpha_{i}=1$, $\partial m_{i}∕\partial t$ is proportional to both $m_{i}$ and $1-m_{i}$, and $S_{i}$ individuals give the same relative importance to both mask-wearers and non-mask-wearers: for a unit increase in the information aggregate, the highest increase in $m_{i}$ occurs when $m_{i}\;=\;0.5$, i.e., when half of the $S_{i}$ individuals have engaged in prophylactic behavior. For general $\alpha_{i}$ values, the highest increase in $m_{i}$ for a unit increase in $\eta_{i}$ occurs when $m_{i}={\left(1+\alpha_{i}\right)}^{-1∕\alpha_{i}}$. It appears that for $\alpha_{i}\in (0,1)$, the highest increase in $m_{i}$ occurs when $m_{i}<0.5$ (weak influence of in-group non-prophylactic behavior), and for $\alpha_{i}>1$, the highest increase in $m_{i}$ occurs when $m_{i}>0.5$ (strong influence of in-group non-prophylactic behavior). Hence, the larger $\alpha_{i}$, the larger impact in-group non-prophylactic behavior will have, slowing down engagement in prophylactic behavior.

Since $m_{i}$ depends on time only through the information aggregate $\eta_{i}$, we can interpret Equation (5) as a differentiation in chain and write $\partial m_{i}∕\partial \eta_{i}=m_{i}\left(1-m_{i}^{\alpha_{i}}\right)∕\alpha_{i}$, which appears to be Richards growth equation [38] with intrinsic growth rate equal to one. Solving for $m_{i}$ yields the generalized logistic curve:

(6a) $$m_{i}={\left[1+\text{exp}\;\{\delta_{i}-\eta_{i}\}\right]}^{-1∕\alpha_{i}}$$

where $\delta_{i}$ is a constant related to the proportion $m_{i0}\;\in \;(0,1)$ of $S_{i}$ susceptibles who would hold a high prophylactic attitude even in the absence of any evidence of disease (i.e., when $\eta_{i}=0$, which happens for t≤τ) by

(6b) $$\delta_{i}=\log\left(m_{i0}^{-\alpha_{i}}-1\right).$$

**FIGURE 1:**
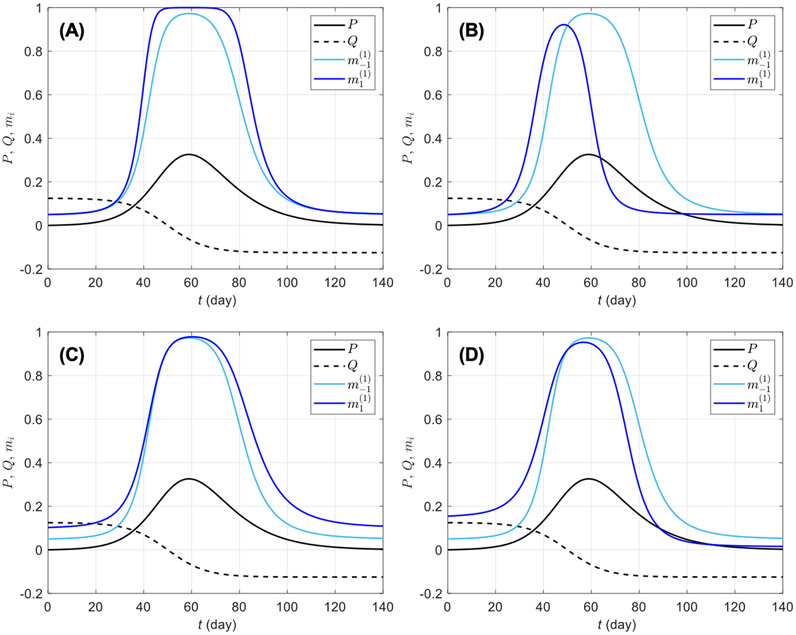
Effects of different information weights on prophylactic proportion $m_{i}^{\left(\alpha_{i}\right)}(i=-1,1)$ for various parameter values. The light blue curve repeated on all plots corresponds to a response to prevalence only, i.e., only the coefficient $a_{i}=20$ is non-zero in Equation (4c); the dark blue curves on plots **(A–D)** correspond to combinations of $a_{i}=20$ with $b_{i}=50$ **(A)**, $c_{i}=150$ **(B)**, $d_{i}=50$ **(C)**, and $e_{i}=10$ **(D)**. The prevalence P (solid black) and the rate of change Q (dashed black) are respectively given by Equations (4a, 4b), $I_{d}$ is given by Equation (3), $\rho_{d}=1∕14$, and the daily number of new detected cases is given by the logistic curve $C(t)=N_{0}e^{v_{t}}\;∕\;{\left(1+e^{v_{t}}\right)}^{2}$ with $N_{0}=100,000$, $v_{t}=\left(t-50\right)∕8$. The proportion of prophylactic individuals in disease-free conditions is $m_{i0}=0.05$ and the in-group behavior parameter is $\alpha_{i}=1$.

Figure 1 shows the prophylactic proportion $m_{i}$ as a function of time for a few selected parameter values, with a perceived disease prevalence P varying from zero to 33%. It appears that the coefficients $b_{i}$, $c_{i}$, $d_{i}$, and $e_{i}$ in Equation (4c) have distinct effects on $m_{i}$ and capture different reactions of susceptibles to disease risk. For instance, $b_{i}$ and $d_{i}$ can be described as response acceleration parameters for high and low prevalence values, respectively. Indeed, a susceptible group more responsive to large prevalence values than to low prevalence values corresponds to $b_{i}\;>\;0$ (Figure 1A), whereas $d_{i}\;>\;0$ corresponds to groups more responsive to low prevalence values than to high prevalence values (Figure 1C). Similarly, $c_{i}>0$ corresponds to groups where engagement in prophylactic behavior is stronger (Figure 1B), and $e_{i}>0$ to groups where engagement in prophylactic behavior is earlier (Figure 1D), but in both cases, disengagement also happens early, once the number of new detected case starts dropping (Q<0).

**FIGURE 2:**
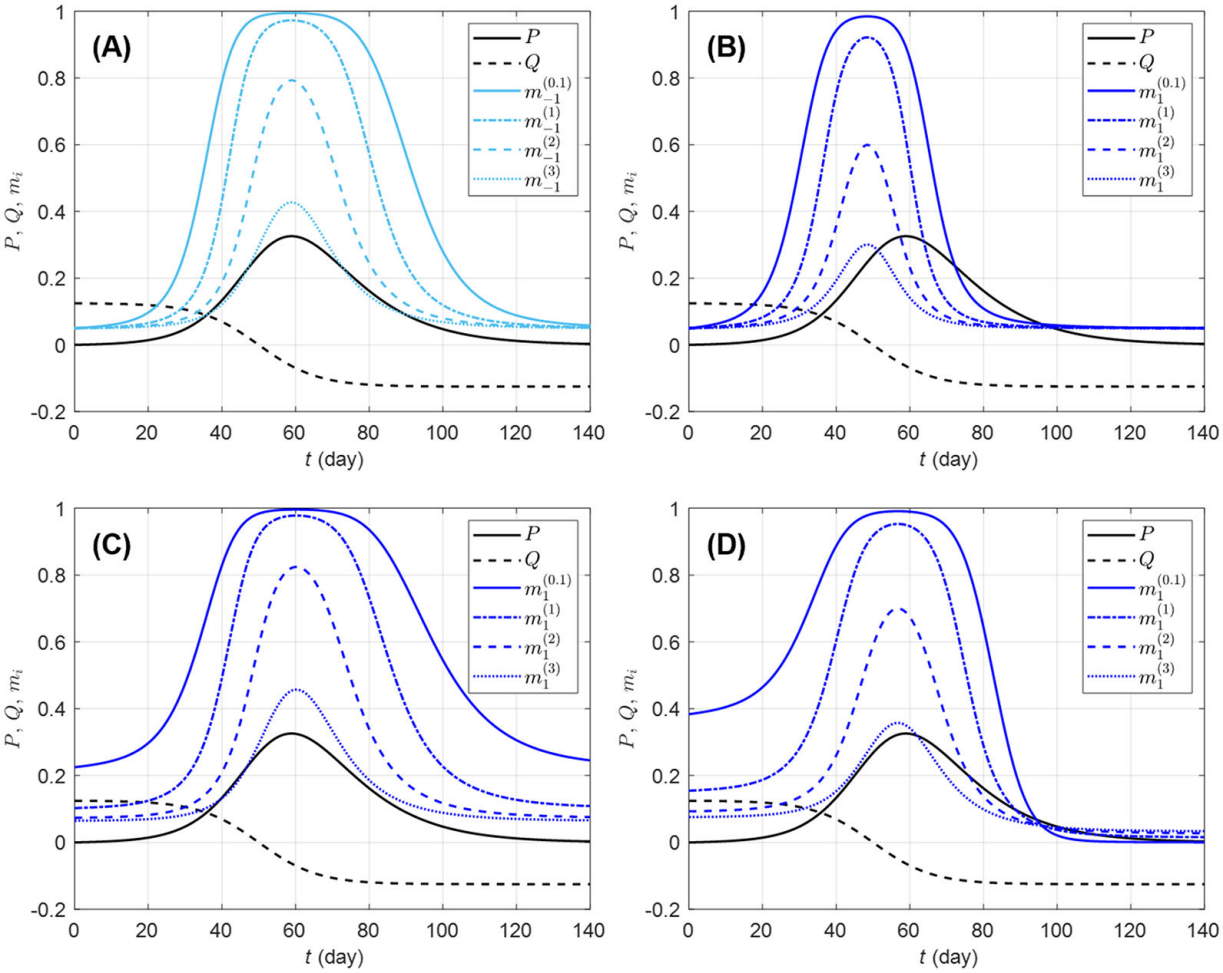
Effects of different in-group behavioral response on prophylactic proportion $m_{i}^{\left(\alpha_{i}\right)}(i=-1,1)$ for various parameter values. The solid light blue curve on graphic **(A)** corresponds to a response to prevalence only, i.e., only the coefficient $a_{i}=20$ is non-zero in Equation (4c); the solid dark blue curves correspond to combinations of $a_{i}=20$ with $c_{i}=150$ **(B)**, $d_{i}=50$ **(C)**, and $e_{i}=10$ **(D)**. All the solid blue curves have in-group behavior parameter $\alpha_{i}=1∕10$ (strong effect of in-group prophylactic behavior). On each plot, the dashed-dotted blue curve corresponds to $\alpha_{i}=1$ (neutral in-group prophylactic behavior), the dashed blue curve to $\alpha_{i}=2$ (strong effect of in-group non-prophylactic behavior), and the dotted blue curve to $\alpha_{i}=3$ (stronger effect of in-group non-prophylactic behavior). The prevalence P (solid black) and the rate of change Q (dashed black) are respectively given by Equations (4a, 4b), $I_{d}$ is given by Equation (3), $\rho_{d}=1∕14$, and the daily number of new detected cases is given by the logistic curve $C(t)=N_{0}e^{v_{t}}\;∕\;{\left(1+e^{v_{t}}\right)}^{2}$ with $N_{0}=100,000$, $v_{t}=\left(t-50\right)∕8$. The proportion of prophylactic individuals in disease-free conditions is $m_{i0}=0.05$.

The in-group behavior parameter $\alpha_{i}$ allows additional flexibility in $m_{i}$ by controlling how the aggregated information is jointly used with how many individuals are already behaving prophylactically in a susceptible group. Whereas $b_{i}$ and $d_{i}$ can be viewed as parameters inducing behavioral response acceleration with respect to change in risk information (P and Q, respectively), $\alpha_{i}$ is an intrinsic acceleration/deceleration parameter, i.e., the acceleration of $m_{i}$ happens not because of change in risk information, but rather in response to the current (low or high) value of $m_{i}$ itself. Figure 2 shows $m_{i}$ curves for a few selected parameter values. It can be observed that *ceteris paribus*, a larger $\alpha_{i}$ value, implies an overall weaker prophylactic behavioral response. Indeed, the derivative of $m_{i}$ with respect to $\alpha_{i}$, given by

(7) $$\frac{\partial m_{i}}{\partial \alpha_{i}}=-\frac{m_{i}}{\alpha_{i}}\left[\log\left(\frac{m_{i}}{m_{i0}}\right)+m_{i}^{\alpha_{i}}\left(1-e^{-\eta_{i}}\right)\log\;(m_{i0})\right],$$

is negative ($m_{i}$ decreases with $\alpha_{i}$) for $\eta_{i}>0$ (i.e., when prophylactic proportion is above the disease-free level $m_{i0}$). However, if information aggregate reaches zero ($\eta_{i}=0$), $\partial m_{i}∕\partial \alpha_{i}$ (Equation 7) vanishes, and if $\eta_{i}$ becomes negative, $\partial m_{i}∕\partial \alpha_{i}>0$. This happens around t=96 days in Figure 2D, where $e_{i}\;>\;0$ (i.e., in a population where Q is given much attention) allows $\eta_{i}<0$ after Q becomes negative, and as the epidemic dies out (P→0), Q dominates the information aggregate: the ordering of prophylactic proportions switches such that a lower $\alpha_{i}$ value corresponds to a weaker prophylactic behavioral response for $\eta_{i}<0$. This can be interpreted as a return of the early engagement of a group with $e_{i}>0$ (Figure 2D, $m_{1}^{\left(0.1\right)}$). In this respect, $\alpha_{i}$ appears as a parameter which exaggerates behavioral response regardless of the sign of information aggregate. But as the disease-free prophylactic proportion $m_{i0}$ is typically low ($m_{i0}=0.05$ in Figure 2), there will generally be less room for this exaggeration when the epidemic dies out ($\eta_{i}<0$) than at disease outbreak ($\eta_{i}>0$).

### 2.4 Contact and transmission rates

We assume for simplicity that the detected infectious individuals are isolated (e.g., hospital and home) and do not mix actively with other classes. Using the “quarantine-adjusted” incidence mechanism [46] yields the force of infection $\lambda_{i}$ (the average number of adequate contacts of one $S_{i}$ susceptible person with infectives per unit time):

(8a) $$\lambda_{i}(t)=\frac{\beta_{ia}(t)I_{a}(t)+\beta_{is}(t)I_{s}(t)}{N(t)-I_{d}(t)}$$

where $\beta_{ia}$ and $\beta_{is}$ are rates of effective contacts with asymptomatic and symptomatic infectious individuals, respectively. The effective contact rate $\beta_{ij}$ depends on a baseline contact rate $\beta_{0}$ (possibly restricted by public health policies), the prophylactic attitude of $S_{i}$ susceptibles, the average efficiency κ∈(0,1) of prophylactic behaviors in reducing transmissions, and the probability $\phi_{j}\in (0,1)$ of disease transmission on contact with $I_{j}$ infections:

(8b) $$\beta_{ij}(t)=\beta_{0}\left[1-\kappa m_{i}(t)\right]\phi_{j}.$$

### 2.5 The behavior-disease dynamics model

**FIGURE 3:**
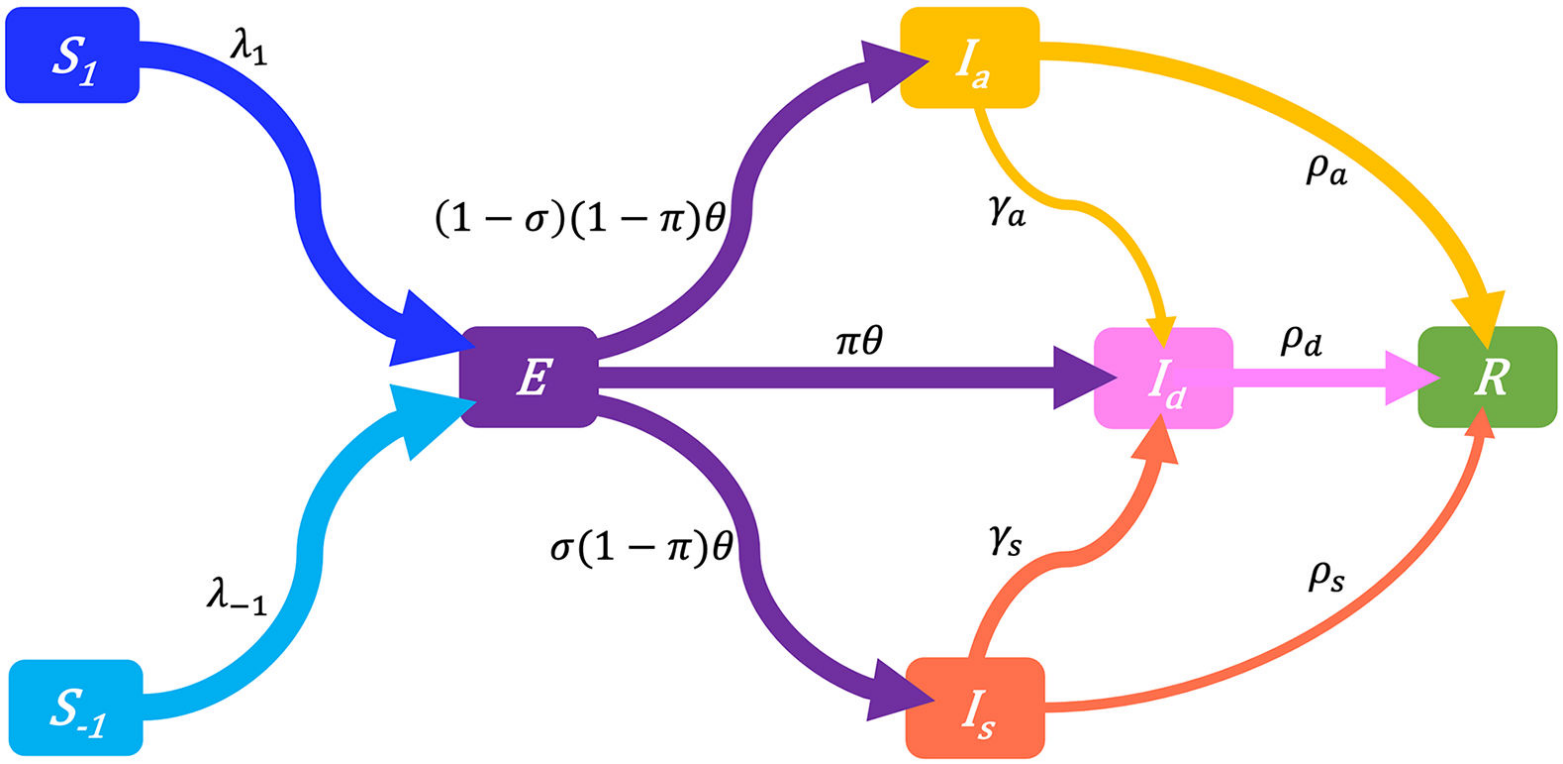
Flow-chart of a Behavior-SEIR dynamics model showing the flow of humans between different compartments. The susceptible population is distinguished in individuals with low standard of evidence ($S_{-1}$) and individuals with high standard of evidence ($S_{1}$). The classes E, $I_{a}$, $I_{s}$, $I_{d}$, and R denote respectively the exposed, the asymptomatic infectious, the symptomatic infectious, the detected infectious, and the removed (recoveries) populations. Recruitment (births and net immigration) and deaths (natural and disease-related) are assumed negligible relative to the population size. The parameters of the model are described in Table 1.

For a target population, we consider a period of study short enough for both disease-related death and natural demographic rates (births, net immigration, and deaths) to be negligible relative to the total population size N (assumed large but finite). As a result, the population size in Equation (1a) remains constant and equal to an initial size $N_{0}\;=\;N(0)$. Joining the behavior and disease dynamics mechanisms described in Sections 2.3, 2.4 gives the Behavior-SEIR model depicted on the flow diagram in Figure 3 with parameters described in Table 1. After sufficient contacts with infectious individual(s), a susceptible individual enters an incubation period (class E) lasting 1∕θ time units on average, in a non-infectious state, and without any disease symptom. Some of these exposed individuals are early detected with probability π thanks to contact tracing or systematic tests on target groups, and enter the class $I_{d}$. In the non-early detected exposed group, 100σ% develop symptoms and enter the class $I_{s}$, and 100(1−σ)% remain asymptomatic and enter the class $I_{a}$. Individuals in the class $I_{s}$ are then identified at a high rate $\gamma_{s}$. Thanks to contact tracing or systematic tests on target groups again, some asymptomatic individuals in the class $I_{a}$ are identified at a lower rate $\gamma_{a}$. All exposed individuals eventually recover from the disease, entering the class R (removals).

The Behavior-SEIR dynamics model is described at time t by the following system of non-linear differential equations:

(9a) $${\dot{S}}_{-1}(t)=-\lambda_{-1}(t)S_{-1}(t),$$

(9b) $${\dot{S}}_{1}(t)=-\lambda_{1}(t)S_{1}(t),$$

(9c) $$\dot{E}(t)=\lambda_{-1}(t)S_{-1}(t)+\lambda_{1}(t)S_{1}(t)-\theta E(t),$$

(9d) $${\dot{I}}_{a}(t)=(1-\sigma )(1-\pi )\theta \;E(t)-(\gamma_{a}+\rho_{a})I_{a}(t),$$

(9e) $${\dot{I}}_{s}(t)=\sigma (1-\pi )\theta \;E(t)-(\gamma_{s}+\rho_{s})I_{s}(t),$$

(9f) $${\dot{I}}_{d}(t)=\pi \theta E(t)+\gamma_{a}I_{a}(t)+\gamma_{s}I_{s}(t)-\rho_{d}I_{d}(t),$$

(9g) $$\dot{R}(t)=\rho_{a}I_{a}(t)+\rho_{s}I_{s}(t)+\rho_{d}I_{d}(t),$$

with the non-negative initial conditions $S_{i}(0)\;=\;S_{i0}$, $E(0)\;=\;E_{0}$, $I_{a}(0)=I_{a0}$, $I_{s}(0)=I_{s0}$, $I_{d}(0)=I_{d0}$. and $R(0)\;=\;R_{0}$ such that $N_{0}=S_{-10}+S_{10}+E_{0}+I_{a0}+I_{s0}+I_{d0}+R_{0}$. The proposed model is a system of neutral delay differential equations [37] where the force of infection $\lambda_{i}$ (Equation 8a) depends through $m_{i}$ (Equation 8b) on the information available at time t, precisely P (dependence on state variables) and Q (dependence on first derivatives of state variables), which are delayed by τ time units.

The parameter τ accounts for two potential sources of information delay: (*i*) reporting delay, i.e., the delay between the moment exposed or infectious individuals are detected and isolated from the mixing population, and the moment the number of detected cases is publicized and can be considered by susceptible individuals to assess their risk, and (*ii*) reaction time, i.e., the delay between the moment the number of detected cases is made public and the moment susceptible individuals actually consider the information to adjust their adherence to preventive prophylactic behavior. We focus on reporting delay which can be included in public health policy design [47]. Indeed, official statistics are often reported with a time delay that may arise from a desire for thorough verification [48]. However, reporting delays can produce the dangerous illusion of an improving epidemic situation since the most recent days have the least cases accounted [48]. Including the time delay parameter τ in model (Equation 9) allows to investigate the extent to which reporting delay can affect the evolution of an epidemic through the behavioral response to delayed information. For our simulation experiments (see Section 2.8), we consider information delays ranging from 1 day to 1 week (τ=1, 3, 5, 7 days).

### 2.6 The effective reproductive number

We compute the effective reproductive number based on the Behavior-SEIR model (Equation 9) using the next-generation matrix approach [49]. Starting from any disease-free state $X^{c}={\left(S_{-10},S_{10},0,0,0,0,R_{0}\right)}^{T}$, the basic reproductive number ℛ(0) for system Equation (9) is given by

(10a) $$ℛ(0)={ℛ}_{o}\sum_{i\in 𝒜}\frac{S_{i0}}{N_{0}}(1-\kappa \;m_{i0}),$$

(10b) $${ℛ}_{o}=\beta_{0}(1-\pi )\left(\phi_{a}\frac{1-\sigma }{\gamma_{a}+\rho_{a}}+\phi_{s}\frac{\sigma }{\gamma_{s}+\rho_{s}}\right)$$

where $N_{0}\;=\;S_{-10}+S_{10}+R_{0}\;>\;0$ and ${ℛ}_{o}$ (Equation 10b) is the reproductive number when there is no differential evidentiary group, $R_{0}=0$, and all prophylactic proportions are zero ($m_{i0}=0$). As expected, the basic reproductive number ℛ(0) (Equation 10a) depends on both the distribution of the population between evidentiary groups, and the prophylactic proportion of each group when no disease evidence is available (both perceived disease prevalence P and rate of change Q are zero). Along an epidemic, the effective reproductive number is then given by

(10c) $$ℛ(t)=\sum_{i\in 𝒜}\frac{S_{i}(t)}{S(t)}{ℛ}_{i(t)}\;\;\;\text{with}$$

(10d) $${ℛ}_{i}(t)={ℛ}_{o}\frac{S(t)}{N_{0}-I_{d}(t)}\left[1-\kappa \;m_{i}(t)\right]$$

where ${ℛ}_{i}(t)$ (Equation 10d) is the effective reproductive number for $S_{i}$ susceptibles such that ℛ(t) is the average of ${ℛ}_{i}(t)$ over all groups of susceptibles.

We would normally conduct here a stability analysis of the model, including the bifurcation diagram for interesting model parameters such as the time delay τ and the level of protection κ. However, these investigations are out of the scope of this conceptual analysis which focuses on the behavioral response to an outbreak and its impact on the dynamic of an epidemic. Such investigations can however be carried out after extending the model to represent more realistic scenarios, including, for instance, vital rates (births and immigration, natural deaths, and disease-related death), flow between susceptible compartments ($S_{-1}$ and $S_{1}$), and immunity lost (flow from R back into $S_{-1}$ and $S_{1}$).

### 2.7 Epidemic severity measures

To allow for comparison between various epidemic scenarios, we define some measures to quantify epidemic severity and overall disease dynamic.

- The number $n_{w}$ of epidemic waves.
  When the maximum number of detected cases ($C_{t}$) over the study period is less than one plus the initial number of infected individuals in the population at time t=0, we consider that the disease dies out and there is no epidemic wave ($n_{w}=0$). Otherwise, we have $n_{w}\geq 1$. For $n_{w}\geq 1$, to cut up the epidemic period based on observable epidemiological data ($C_{t}$) and identify epidemic waves, we consider a wave detection algorithm based on five properties imposed upon the height of waves, and the troughs between waves: (*i*) the number of detected cases at the trough between two successive waves is <500, (*ii*) the duration between a trough and the preceding peak is 5 days or more, (*iii*) an epidemic wave lasts at least 1 week, (*iv*) the prominence of the peak of a wave is 10 or more detected cases (per day) above the surrounding valley (specifically the highest of the left and right troughs), and (*v*) the prominence of the peak represents at least 50% of the peak height. The algorithm is a modification [addition of properties (*i*) and (*ii*)] of Harvey et al. [50]’s epidemic wave identification algorithm. The wave detection algorithm is implemented in R freeware [51] (R package *wavefinder* with source available on the Git repository SE-SEIR).
- The overall peak height $H_{p}$ (number of detected cases) and peak time $T_{p}$ (days).
- The time $T_{c1}$ to curb the first outbreak.
  The duration from disease outbreak (ℛ(0)>1) to the first time when the effective reproductive number ℛ (Equation 10c) falls to one:
(11) $$T_{c1}=\underset{t}{\text{argmin}}\left\{ℛ(t)=1\right\}.$$
- The final epidemic size $F_{T}$.
  For a given time horizon T, the final epidemic size is given by $F_{T}=1-S_{T}∕N_{0}$, where $S_{T}$ is the total number of susceptible individuals at time t=T and $N_{0}$ is the total (initial) population size.

### 2.8 Simulation experiment

We carried out a simulation experiment to explore combinations of model parameters related to disease transmission ($\beta_{0}$), early detection of exposed individuals (π), delayed acquisition or reaction to risk information (τ), behavioral reaction ($a_{i}$, $b_{i}$, $c_{i}$, $d_{i}$, and $e_{i}$), in-group behavior ($\alpha_{i}$), and efficiency of prophylactic behaviors (κ) that lead to patterns where the course of an epidemic depends more or less on differences between risk perception and related feedback loops.

#### 2.8.1 Simulation design

We first considered a variety of basic behavioral response profiles including a reference population (*0*) responsive to prevalence only, and the four profiles of populations in Figures 1A-D: profile *0* corresponds to $a_{i}=20$ ($b_{i}=c_{i}=d_{i}=e_{i}=0$), profile A corresponds to $a_{i}=20$ and $b_{i}=50\;(c_{i}=d_{i}=e_{i}=0)$, profile B to $a_{i}=20$ and $c_{i}=50\;(b_{i}=d_{i}=e_{i}=0)$, profile C to $a_{i}=20$ and $d_{i}=50\;(b_{i}=c_{i}=e_{i}=0)$, and profile D to $a_{i}=20$ and $e_{i}=10\;(b_{i}=c_{i}=d_{i}=0)$. These five profiles are homogeneous with respect to behavioral reaction to risk information. We then included heterogeneous populations obtained as combinations of the reference profile (half of the population) with one of profiles A−D. For each profile, we varied other model parameters including $\beta_{0}$, π, τ, κ, and $\alpha_{i}$ (see parameter values in Table 1). Since $\alpha_{i}$ is group specific, we have the between-group difference $\Delta \alpha =\alpha_{-1}-\alpha_{1}$, which measures heterogeneity in in-group pressure.

For each simulation setting, we solved system (Equation 9) using the function *dede* from the R package *deSolve* [52] (code in the R package *BSEIR* with source available on the Git repository SE-SEIR), and recorded the number of epidemic waves up to T=1,000 days after the first outbreak ($n_{w}$), the overall peak time ($T_{p}$) and height ($H_{p}$), the time to curb the first epidemic wave ($T_{c1}$), and the final epidemic size ($F_{1,000}$).

#### 2.8.2 Statistical analyses

To summarize simulation results, we computed descriptive statistics for epidemic severity measures in R, and fitted generalized linear models [53] to the number of secondary epidemic waves (Poisson regression) and the epidemic peak size (gamma regression) as functions of population response profiles and the varied model (Equation 9) parameters (see descriptive statistics in Supplementary Table S1). We also fitted a one-inflated beta regression model [54] to the final epidemic size ($F_{1,000}$) using the R package *gamlss* [55]. For each model, we checked goodness-of-fit using a $\chi^{2}$-test on residual deviance, and evaluated the explanatory power of the model using a deviance based pseudo-$R^{2}$ [56]. Supplementary Table S2 provides descriptive statistics for the different epidemic severity measures over all simulation scenarios.

## 3 Results

### 3.1 Epidemic severity across types of responses

Our simulation results indicate important variations in epidemic severity measures between levels of in-group pressure. Indeed, an increase in in-group pressure for pro-prophylactic behavior (decreasing $\alpha_{i}$) increases the average number of secondary waves, but decreases the epidemic peak size and final size (Table 2).

Table 3 shows summaries of epidemic severity measures comparing populations with the reference response profile *0* and profiles A−B, averaged over all levels of in-group pressure ($\alpha_{i}\in \{0.1,1,2,3\}$). All investigated parameter settings result into an epidemic, the disease quickly dying out in only 1% of settings with the homogeneous profile B (i.e., populations with a reaction to rate of change Q proportional to perceived prevalence P). When there is an epidemic, one secondary epidemic wave occurs in 9% of settings with the reference profile *0*. Secondary epidemic waves are the most likely (19%) under profile A [homogeneous populations with a strong (quadratic) reaction to P only], and the least likely (2%) under profile B. The epidemic peaks after about 64 days on average in populations responsive to P only (profile *0*) to ~3,209 new reported cases. Slightly lower average peak time and size result from a stronger reaction to P (3,050 case after 62 days for profile A) or an additional response to Q (3,126 cases after 60 days for profile D). Ignoring the sign of Q increases the average peak time to about 72 days (profile C) while decreasing the peak size to ~32,748 cases, with intermediate result for profile B (2,840 cases after 66 days). The final epidemic size is typically large, 91% on average, under profile *0* (and C). A stronger reaction to P (profile A) slightly reduces the final size to 90% whereas an additional response to Q (profiles B and D) increases the final size to 94%. In summary, a stronger reaction to P essentially leads to more secondary waves whereas an additional response to Q hastens the epidemic peak and increases the final size, with lower peak sizes in both scenarios. A response to Q proportional to P also increases average final size, but delays the peak (with a lower size).

**FIGURE 4:**
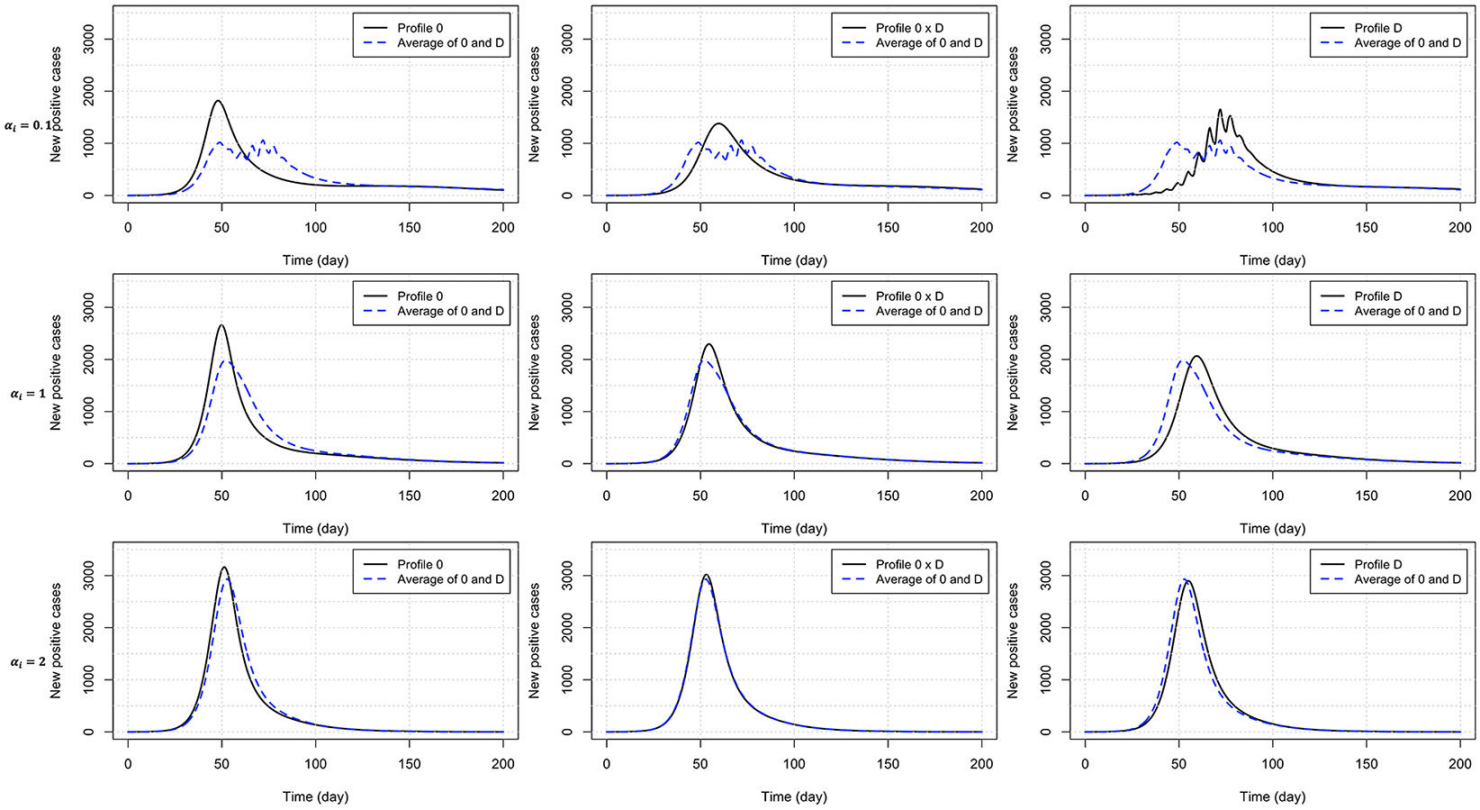
Number of new positive cases for homogeneous populations responsive to prevalence only (profile 0) or both prevalence and the rate of change of new positives (profile D), and an heterogeneous population (half profile 0 and half profile D) with pro-prophylactic behavior ($\alpha_{i}=0.1$), neutral ($\alpha_{i}=1$), or non-prophylactic behavior ($\alpha_{i}=2$) in-group pressure. The dashed blue curve indicates for each row (each $\alpha_{i}$, value) the average of the numbers of new positive cases for profiles 0 and D. Disease-related parameters have their default (bold) values in Table 1.

Heterogeneity in the behavioral reaction to risk information generally leads to intermediate results half way between the two combined homogeneous profiles (Table 3). Exceptions include the time to curb the first epidemic wave (larger than expected) under profiles 0×C and 0×D, and the peak time (larger than expected) and size under profile 0×D (smaller than expected). Figure 4 shows the timely number of new positive cases under profiles *0*, D, and 0×D, exhibiting an interactive effect between in-group pressure and heterogeneity. Indeed, it appears that in an heterogeneous pro-prophylactic behavior population (Figure 4, α=0.1) where half of individuals are responsive to P and the other half is additionally responsive to Q, the epidemic peak size (1,380 new detected cases) is smaller than in homogeneous populations with profile *0* (1,819 new detected cases) or D (1,653 new detected cases).

When we restrict attention to populations where in-group pressure is neutral ($\alpha_{i}=1$), we observe that a stronger reaction to P leads to one secondary epidemic wave in 24% of settings, as opposed to 19% across $\alpha_{i}$ values (Supplementary Table S3). Similarly, an additional response to Q increases the peak time and does not increase the final epidemic size (90% under profile D), unless the reaction to Q is proportional to P (93% under profile B). These discrepancies point out to important variations between levels of in-group pressure.

The results of fitted models shown in Table 4 corroborate our observations for the number of secondary waves (model coefficient estimates < 0 for $\alpha_{-1}$ and α), the peak size, and the final epidemic size (coefficient estimates > 0 for $\alpha_{-1}$ and Δα). It also appears that among the profiles A−D, only profile B (population with a response to the rate of change proportional to prevalence) leads to a decrease in the number of secondary epidemic waves and an increase in the final epidemic size, as compared to the reference profile *0*. In addition, the model results indicate that for an heterogeneous population, the expected number of secondary epidemic waves or the expected final epidemic size is intermediate between the outcomes for the two corresponding homogeneous populations, except when half the population has profile D. For the latter, after controlling for disease dynamic and in-group pressure parameters, the expected number of secondary epidemic waves is 15.9% (100 × 0.159) higher for the homogeneous profile D, but 31.8% higher for the heterogeneous profile 0×D, as compared to an homogeneous profile *0*.

The fitted models also indicate that a 1-day increase in risk information delay (τ) leads to a 2.6% average increase (100 × 0.026) in the average odd ratio for a random individual to get infected over the course of the epidemic, a 3.3% increase in the overall peak size, and a 6.5% decrease in the number of secondary waves (Table 4). A discussion of the biological interpretation of these statistics is provided in Section 4.1. As for other varied model parameters ($\beta_{0}$, π, and κ), apart from in-group pressure, the variations of the number of secondary waves are mostly driven by the level of protection by prophylactic behavior, to the extent that a 1%-point increase in κ results into a 15.5% increase in the number of secondary waves. In other words, if 80% level of protection yields one epidemic wave on average, then increasing the level of protection by 10% (i.e., from 80 to 90%) results in an expected 2.55 waves (1 + 1 × 0.155 × 10). Both the peak size and the final epidemic size are mainly determined by the baseline transmission rate ($\beta_{0}$) and the probability of early detection of exposed individuals (π).

### 3.2 Epidemic severity across levels of prophylactic protection

**FIGURE 5:**
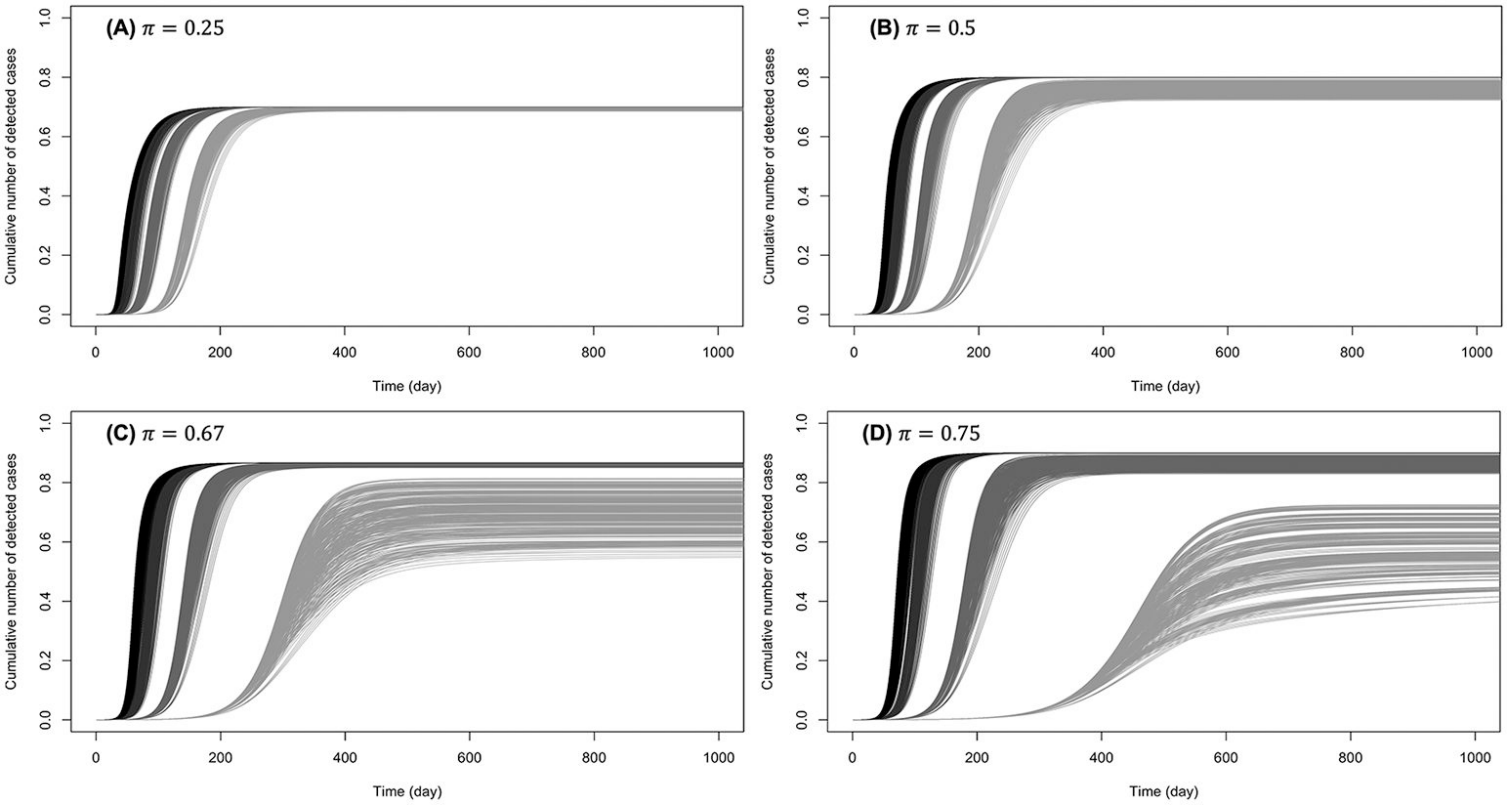
Cumulative number of detected cases when the efficiency of prophylactic behavior is medium: κ∈{0.5,0.6}. The curves on **(A)** correspond to low early detection probability (loose disease surveillance mechanism), **(B)** medium early detection probability, **(C)** high early detection probability, and **(D)** very high early detection probability. For each probability of early detection of exposed individuals (π), the rightmost gray curves correspond to the lowest baseline disease transmission rate ($\beta_{0}=0.5$) and the dark curves (leftmost) correspond to the largest transmission rate ($\beta_{0}=3$). More or less gray curves have intermediate transmission rates ($\beta_{0}=1,2$).

**FIGURE 6:**
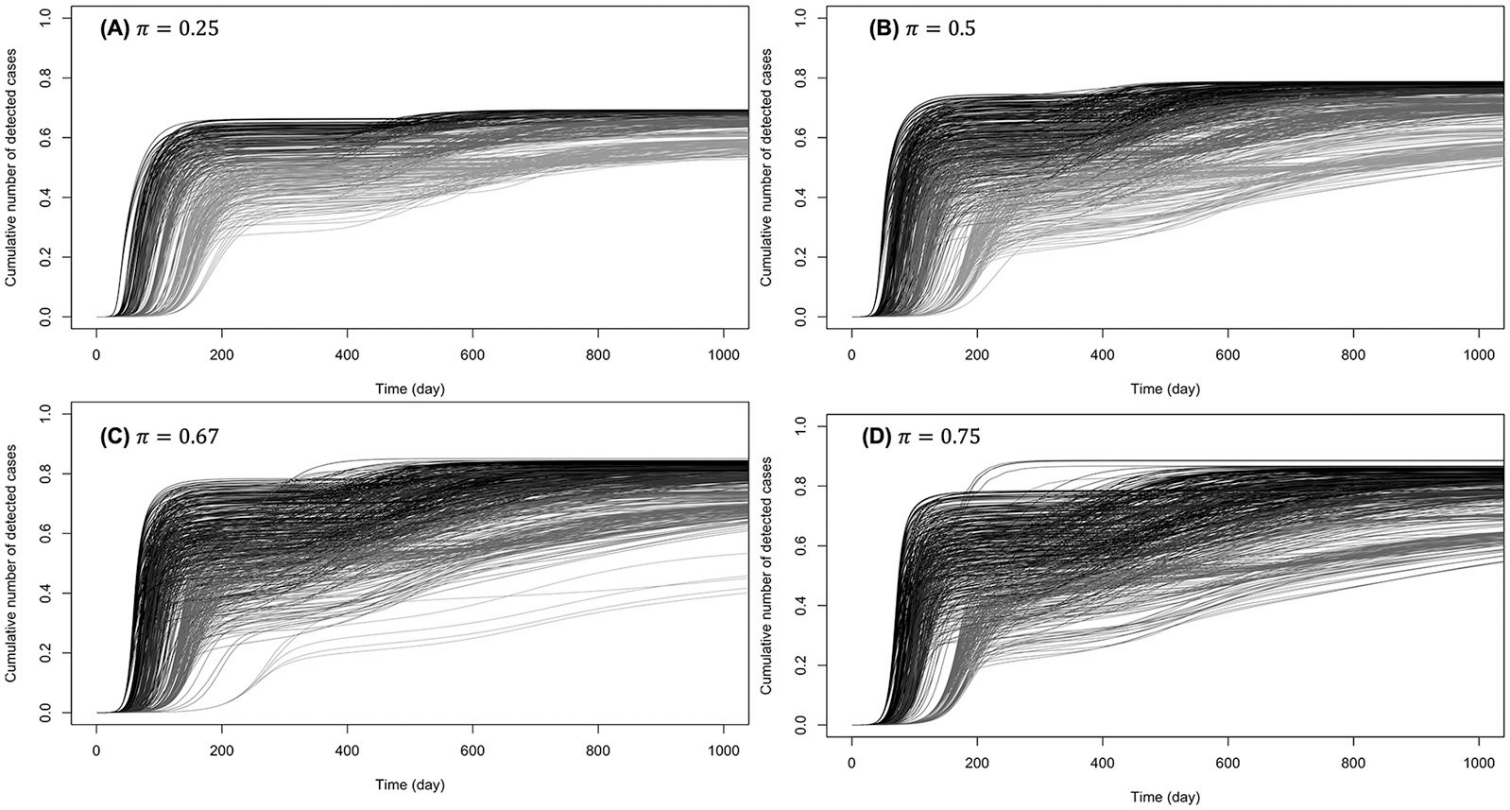
Cumulative number of detected cases when there are one or two secondary waves with (almost) perfect protection from prophylactic behavior: κ∈{0.95,1}. The curves on **(A)** correspond to low early detection probability (loose disease surveillance mechanism), **(B)** medium early detection probability, **(C)** high early detection probability, and **(D)** very high early detection probability. For each probability of early detection of exposed individuals (π), the gray curves (rightmost) correspond to the lowest baseline transmission rate ($\beta_{0}=0.5$) and the dark curves (leftmost) correspond to the largest baseline transmission rate ($\beta_{0}=3$).

Figures 5, 6 show the cumulative number of cases detected up to 1,000 days after the outbreak for various values of κ, π, and $\beta_{0}$. For κ∈{0.5,0.6}, no secondary epidemic wave was observed (Figure 5). It appears that if the disease surveillance mechanism for early detection and removal is loose (π=0.25), disease dynamic is barely sensitive to behaviors (Figure 5A). When disease surveillance is more effective (π≥0.5), disease dynamic (peak and observed final size) becomes more sensitive to behaviors (Figures 5B-D), especially when the baseline disease transmission rate is low ($\beta_{0}=0.25$).

When prophylactic behavior offers (almost) perfect protection (κ∈{0.95,1}), secondary epidemic waves (one or two) were observed in 28% of these settings. Figure 6 shows the cumulative numbers of detected cases when there are secondary waves (see Supplementary Figure S1 for unique wave scenarios). It can be observed that at such high levels of protection by prophylactic behavior, disease dynamic is highly sensitive to behavioral changes, even when the baseline disease transmission rate is high ($\beta_{0}=3$).

When the efficiency of prophylactic behavior is between these two extremes (κ∈{0.75,0.9}), secondary epidemic waves (one or two) were observed in 4% of these settings (Supplementary Figures S2, S3): with these levels of protection by prophylactic behavior, disease dynamic is already sensitive to behavioral changes, especially when the baseline disease transmission rate is $\beta_{0}\;<\;2$, and the higher the probability of early detection (π), the higher the number of secondary epidemic waves.

## 4 Discussion

### 4.1 Main contributions

In this study, we introduce a new behavior-disease compartmental model where risk perception is a function of both perceived disease dynamic and an interpretation domain (in-group pressure). That is, the risk information derived from disease dynamics can include predicted incidence (as expressed by the rate of change Q of new positive cases) in addition to historical incidence (as expressed by the perceived prevalence P given the disease surveillance system), and the actual risk perceived by an individual can arise not only from disease dynamics but also from the pro/non-prophylactic behavior of others in the individual’s social group.

Under the social influence-based model which uses true disease incidence as risk information, Tyson et al. [14] found that populations more responsive to risk information can experience more severe epidemics in terms of final size and undergo multiple epidemic waves, although the epidemic peak sizes will be smaller.

We observe similar trends when matching increase in social influence with increase in pro-prophylactic behavior in-group pressure. Considering populations responsive to historical risk information, our results regarding the number of epidemic waves and the peak size are consistent with Tyson et al.’s [14] findings, i.e., a stronger reaction to perceived disease prevalence produces more epidemic waves but smaller peak size. Indeed, a strong reaction to prevalence results in transient prophylaxis that slows down and stops disease progression (smaller peak) while many susceptible individuals are still in the population. Low disease prevalence then leads to relaxation of prophylactic behaviors and subsequently to a new outbreak (multiple waves).

A new aspect of behavior-disease dynamic captured by our model is the importance of response profiles. For instance, we considered two kinds of populations, ones that are only responsive to historical risk and ones that are additionally responsive to predicted risk. For risk predicting populations, the epidemic peak size does not monotonically decrease with increasing pro-prophylactic in-group pressure (the epidemic peak size is smallest when in-group behavior is neutral). In other words, in the context of risk information overload [36], in particular, when disease evolution curves are overly discussed on mass media and social media, stronger social influence or in-group pressure can lead to more severe epidemic outcome, at least as measured by the epidemic peak size.

Our results also indicate that erratic disease evolution curves can be explained by strong behavioral response to predicted disease curves shown on mass media or in social media. For a population paying attention to predicted risk information, a strong response reduces the time scale of the chain of reactions that leads to multiple epidemic waves, giving rise to very fast oscillations in the observed disease incidence curve (see Figure 4), both as the epidemic establishes (before peak) and as the epidemic is waning. This risk-prediction feature also makes our model quite different from others where each epidemic peak is necessarily followed by an almost disease-free interval before emergence of the following peak [14, 57]. Because of this, which is rather frequent is real epidemic data [50], our model requires a wave delimitation method to identify epidemic waves. Also note that, unlike in Aziz-Alaoui et al. [2], our model targets a short-term dynamic, and epidemic waves in our model framework are not related to immunity lost (no flow from R back to $S_{i}$ in Figure 3) but fully generated by the behavioral response to the outbreak.

Our model allows us to explore the impact of heterogeneity in the behavioral response of a population to disease risk information. In general, two-group heterogeneity in response profile (pro- vs. contra-prophylactic behavior in-group pressure, or responsiveness to predicted risk or not) leads to an intermediate epidemic outcome as compared to the two sub-populations evolving separately. One interesting finding is that under strong pro-prophylactic in-group pressure, a population consisting of two same sized sub-populations where only one group is exposed/responsive to predicted risk information can experience a less severe epidemic as measured by peak size.

Finally, we investigated the effect of delayed risk information on the severity of an epidemic in our model framework. Our simulation experiments indicate that delayed risk information slows down the behavioral response to the progression of the epidemic, contributing to a more severe epidemic outcome, i.e., larger peak and final size. For an individual with a 50% average risk to get infected in a 1-day reporting delay context, the risk to get infected becomes 54% if the reporting delay is 1 week (6 days increase). Although a 4% increase may appear small at an individual level, it would represent 4,000 more infections, given the population size in our simulations (community of 100,000 individuals). Similarly, for an epidemic that peaks to 1,500 new detected cases (see, e.g., Figure 4) under a 1-day reporting delay scenario, a reporting delay of 1 week would result into 50 additional detected cases on the peak day. These simulated results are in accordance with the work of Gutierrez et al. [47], who found that the COVID-19 epidemic in Mexico progressed much faster when delays are larger, resulting in more severe epidemic outcomes (larger death peak size and cumulative death toll). A viable solution for policymakers to reduce information delay-related increase in epidemic severity is to use a nowcasting technique to adjust the daily number of confirmed new cases for delayed reporting [48], especially in populations reactive to predicted risk, since delayed risk information creates an illusion of a downward trend. We must remind the reader, however, that our primary interest is to do a conceptual analysis and none of these results should be taken literally without empirical validation.

### 4.2 Limitations

Although the proposed model framework is quite general for coupling risk tolerance and disease dynamics, we have in our presentation limited attention to populations with relatively simple structures. In this section, we highlight and discuss the most important assumptions that may strongly affect our conclusions, and explore some potential routes to their relaxation. The prophylactic proportion defined in Equation (6) makes the strong assumption that risk information derived from in-group behavior is of no use if there is no change in risk information aggregate. However, in-group behavior can lead to change in prophylactic behavior even if risk information is constant, i.e., people can change behavior by inferring the need to engage (or the appropriateness of disengaging) based on how many individuals are already (or are still) behaving prophylactically, not because the risk has increased or decreased but because there is extant risk and in-group pressure has changed the risk perception.

Another strong assumption in Equation (6) is that the evolution of prophylactic behavior does not depend on how long people have already been engaging in prophylactic behavior, and how strong their engagement was during that time. However, prophylactic behavior is often subject to fatigue. Indeed, preventive behavior fatigue during secondary epidemic waves of a disease has been particularly well-documented since the COVID-19 pandemic event [58-75]. Failing to account for prophylactic behavior fatigue in behavior-disease model likely introduces bias in model outputs.

To account for the above two limitations of Equation (6), we propose to extend Equation (5) describing change in the proportion of prophylactic individuals in a susceptible group i as:

(12) $$\frac{\partial m_{i}}{\partial t}=\left[m_{i}\frac{1-m_{i}^{\alpha_{i}}}{\alpha_{i}}\right]\left[\frac{\partial \eta_{i}}{\partial t}+\omega_{i}\eta_{i}\right](1-f_{i})$$

where $\omega_{i}\;\geq \;0$ is the weight of “indirectly perceived change in risk” derived from in-group behavior (how informative in-group behavior is about perceived risk) and $f_{i}\in [0,1]$ represents prophylactic fatigue in group i. It follows from Equation (12) that Equation (6a) can be generalized as:

(13a) $$m_{i}={\left[1+\text{exp}\left\{\delta_{i}-\eta_{i}\;(1-f_{i})-\zeta_{i}\right\}\right]}^{-1∕\alpha_{i}}$$

(13b) $$\text{with}\;\;f_{i}(t)=1-\text{exp}\left\{-\epsilon M_{i}(t)\right\},\;\;\text{and}$$

(13c) $$\zeta_{i}(t)=\int_{0}^{t}\eta_{i}(u)\left[{\dot{f}}_{i}(u)+\omega_{i}[1-f_{i}(u)]\right]du$$

where we assumed that enthusiasm for prophylactic behavior decays exponentially as experience under disease-related restrictions increases, with ϵ≥0 expressing the extent to which prophylactic behavior is exhausting (ϵ=0 means that there is no prophylactic fatigue over time, $f_{i}=0$), and the variable $M_{i}$ quantifies how much effort the susceptible group i has invested in prophylactic behavior since the epidemic outbreak:

(13d) $${\dot{M}}_{i}=\log\left(\frac{m_{i}^{\alpha_{i}}}{1-m_{i}^{\alpha_{i}}}\right)-\log\left(\frac{m_{i0}^{\alpha_{i}}}{1-m_{i0}^{\alpha_{i}}}\right)$$

and $M_{i}(0)=0$. Note from Equation (13a) that ${\dot{M}}_{i}$ can be rewritten as ${\dot{M}}_{i}=\eta_{i}(1-f_{i})+\zeta_{i}$. Equation (13a) is reduced to Equation (6a) when $\omega_{i}\;=\;0$ and $f_{i}(t)\;=\;0$. To solve system (9) with the prophylactic proportion $m_{i}$ given by Equation (13), we can extend the differential system Equation (9) with Equation (13d) for the pseudo-state variable $M_{i}$, and an additional pseudo-state variable $\zeta_{i}$ whose first derivative is given by the integrand in Equation (13c), i.e., ${\dot{\zeta }}_{i}=\eta_{i}\left[{\dot{f}}_{i}+\omega_{i}\left(1-f_{i}\right)\right]$, which simplifies to

(14) $${\dot{\zeta }}_{i}=\eta_{i}\left(1-f_{i}\right)\left(\epsilon {\dot{M}}_{i}+\omega_{i}\right).$$

However, it appears that prophylactic fatigue, as introduced in Equations (12-14), affects equally engagement and disengagement in prophylactic behavior. A *post-hoc* but inelegant solution to that issue is to modify Equation (12) to have the form in Equation (15):

(15) $$\frac{\partial m_{i}}{\partial t}=\left[m_{i}\frac{1-m_{i}^{\alpha_{i}}}{\alpha_{i}}\right]\left[\frac{\partial \eta_{i}}{\partial t}+\omega_{i}\eta_{i}\right](1-f_{i}g_{i})$$

where $g_{i}\;=\;1$ if $\partial \eta_{i}∕\partial t\;\geq \;0$ and 0 otherwise. We leave the implementation and exploration of such speculations for future work.

Another strong hypothesis of our SEIR framework is the well-mixture assumption, i.e., for each time point, the probability of interaction between two random individuals in the population is uniformly distributed [76]. While a low standard individual might tend to interact more with low standard individuals than high standard individuals, for instance, our model assumes an homogeneous mixture of low standard and high standard groups. More generally, heterogeneity in a population goes beyond risk tolerance groups, and may be related to other factors such as geographical location [77], age [78], and behavioral risk factors [79]. In particular, the mixture of individuals from various geographical regions is generally non-uniform, and explains the large disparities among different geographical locations in the COVID-19 pandemic context [80]. Thus, to be realistic, our model should be extended to include spatial components to reveal or account for the contribution of the spatial structure of individuals to an observed epidemic dynamic. This can be achieved using, for instance, non-autonomous coupling functions between adjacent areas [6], a diffusion process [81], an agent-based infection graph [82], or a location network [83].

Finally, we point out to the possibility of an evolving pathogen, as observed for the COVID-19 pandemic [84]. For a multi-strain pathogen, a realistic epidemic model should account for mutation process occurring during infection of individuals in the population [85, 86]. As such a future direction for our study is to consider many variants of a target disease and investigate behavioral feedback loops as a pathogen strain dominates the population and is then replaced by a new pathogen, appearing through evolutionary process or interactions with adjacent geographical regions.

## 4.3 Conclusion

In this study, we assessed the impact of differential behavioral response profile on epidemic outcomes. Our main contributions for understanding feedback loops between transient prophylaxis and disease dynamic include (*i*) the distinction of historical risk from predicted risk information overly discussed on mass media and social media, and (*ii*) the inclusion of an interpretation domain where the collected risk information is subjected to in-group pressure. It was known that the final size of an epidemic has a non-monotonic relation with the behavioral response of a population to risk information. Our results indicate that this non-monotonicity extends to epidemic peak size as a measure of epidemic severity, in populations under strong in-group pressure.

An obvious future direction is to assess the ability of this model to predict disease dynamics, using real epidemic data (from, e.g., the world health organization, https://covid19.who.int) along with behavioral change data from surveys (e.g., adherence to COVID-19 protective measures [87, 88]). The inclusion of more than two social groups, based, for instance, on age or spatial location may be integral parts for establishing some predictive power of the model. However, our aim here was not predictive, but rather to better understand how to represent assumptions about heterogeneous risk tolerance and in-group pressures, and then in turn study their potential effects on disease dynamics.

## Supplementary Material

Supplementary Material

## Figures and Tables

**TABLE 1:** Description and values of parameters in the behavior-disease model.

Parameter	Description	Values
τ	Time delay (days) of risk information	1, **3**, 5, 7
$a_{i}^{†}$	Main response of $S_{i}$ (susceptibles) to prevalence (P)	20
$b_{i}$	Non-linear response of $S_{i}$ to P (i.e., $P^{2}$)	**0**, 50
$c_{i}$	Responsiveness of $S_{i}$ to the interaction P×Q	**0**, 150
$d_{i}$	Non-linear response of $S_{i}$ to Q (i.e., $Q^{2}$)	**0**, 50
$e_{i}$	Main response of $S_{i}$ to rate of change (Q)	0, **10**
$\alpha_{i}$	Strength of in-group non-prophylactic behavior	0.1, **1**, 2, 3
$m_{i0}$	Prophylactic proportion in the absence of risk evidence	0.05
κ	Average efficiency of prophylactic behavior	$\frac{1}{2}$, $\frac{3}{5}$, $\frac{3}{4}$, $\frac{9}{10}$, $\frac{19}{20}$, 1
$\beta_{0}$	Baseline transmission rate	1/2, **1**, 2, 3
$\phi_{j}^{*}$	Probability of disease transmission by $I_{j}$ infectious	1
1∕θ	Duration of incubation (latent) period (days)	4
π	Early detection probability for exposed individuals	$\frac{1}{4}$, $\frac{1}{2}$, $\frac{2}{3}$, $\frac{3}{4}$
σ	Proportion of symptomatic infectious	1/2
$\gamma_{j}$	Detection rate of $I_{j}$ infectives	$\frac{2}{14\times 5}$, $\frac{4}{14\times 5}$
$\rho_{j}$	Removal rate of $I_{j}$ infectious	$\frac{3}{14\times 5}$, $\frac{1}{14\times 5}$, $\frac{1}{70}$
$S_{i0}$	Initial number of $S_{i}$ susceptibles	49, 998
$E_{0}$, $I_{j0}$	Initial number of exposed and infectious	2, 1, 1, 0
$R_{0}$	Initial number of recovered individuals	0
$N_{0}$	Total population size	100, 000

All parameters take non-negative values.

† i= standard of evidence level defined in Equation (1b).

* j=a (asymptomatic), s (symptomatic), or d (detected) infectives.

For parameters with multiple values (τ, $a_{i}$, $c_{i}$, $d_{i}$, $e_{i}$, $\alpha_{i}$, κ, $\beta_{0}$, π), the default value is bolded.

**TABLE 2:** Summary of epidemic severity measures comparing populations with various levels of in-group pressure ($\alpha_{i}\in \{0.1,1,2,3\}$).

Statistics^†^	Median	Mean	SD	Median	Mean	SD
	$\alpha_{-1}=\alpha_{1}=0.1$			$\alpha_{-1}=0.1$, $\alpha_{1}=1$		
Epidemic?	1.00	1.00	0.06	1.00	1.00	0.05
Nb2. waves	0.00	**0.26**	0.45	0.00	0.20	0.41
Peak time	50.50	67.26	47.88	49.50	66.95	49.11
Peak size	2,158.11	*2,293.38*	1,473.21	2,453.50	2,499.49	1,480.42
Time to curb*	48.50	65.91	50.64	49.50	67.10	51.55
Final size	0.95	*0.88*	0.15	0.96	0.89	0.15
	$\alpha_{-1}=\alpha_{1}=1$					
Epidemic?	1.00	1.00	0.03			
Nb2. waves	0.00	0.12	0.33			
Peak time	47.50	65.44	49.53			
Peak size	2,838.61	2,826.22	1,525.38			
Time to curb*	47.00	65.73	51.47			
Final size	0.97	0.91	0.13			
	$\alpha_{-1}=\alpha_{1}=2$			$\alpha_{-1}=1$, $\alpha_{1}=2$		
Epidemic?	1.00	1.00	0.00	1.00	1.00	0.02
Nb2. waves	0.00	*0.02*	0.15	0.00	0.06	0.25
Peak time	46.00	65.82	52.62	46.50	65.80	50.91
Peak size	3,371.64	**3,295.01**	1,580.77	3,010.69	2,990.65	1,525.65
Time to curb*	45.50	67.00	54.03	47.50	67.12	52.48
Final size	0.99	**0.93**	0.11	0.98	0.91	0.13
	$\alpha_{-1}=\alpha_{1}=3$			$\alpha_{-1}=1$, $\alpha_{1}=3$		
Epidemic?	1.00	1.00	0.00	1.00	1.00	0.00
Nb2. waves	0.00	0.01	0.09	0.00	0.01	0.11
Peak time	45.50	66.36	54.49	45.50	66.07	53.38
Peak size	3,551.96	**3,540.48**	1,619.83	3,459.73	3,385.74	1,587.77
Time to curb*	45.00	68.11	55.92	45.50	67.78	54.75
Final size	1.00	**0.96**	0.08	1.00	0.94	0.09

SD, standard deviation; Nb2. waves, number of secondary waves.

† The statistics are based on n=3,456 simulations across the profiles 0−D, and values of model parameters $\beta_{0}$, π, τ, and κ in Table 1.

* Time to curb = time to curb the first epidemic wave defined in Equation (11).

Italic (bold) figures indicate average values lower (larger) than the reference ($\alpha_{-i}=1$) for homogeneous populations ($\alpha_{-1}=\alpha_{1}$).

**TABLE 3:** Summary of epidemic severity measures comparing populations with profiles A–B to the reference profile 0 under various in-group pressure ($\alpha_{i}\in \{0.1,1,2,3\}$).

Statistics^†^	Median	Mean	SD	Median	Mean	SD
	*0* (Prevalence only, $a_{i}=20$)					
Epidemic?	1.00	1.00	0.00			
Nb2. waves	0.00	0.09	0.29			
Peak time	42.00	63.50	51.53			
Peak size	3,214.47	3,209.03	1,707.75			
Time to curb*	44.00	65.36	52.80			
Final size	0.98	0.91	0.13			
	A ($a_{i}=20$, $b_{i}=50$)			0×A		
Epidemic?	1.00	1.00	0.00	1.00	1.00	0.00
Nb2. waves	0.00	**0.19**	0.39	0.00	0.15	0.35
Peak time	41.50	61.97	4941.50	62.62	50.18	
Peak size	3,007.55	*3,049.14*	1,708.02	3,050.56	3,104.21	1,707.56
Time to curb*	43.50	63.38	50.56	43.50	64.58	51.38
Final size	0.96	0.90	0.14	0.97	0.91	0.14
	B ($a_{i}=20$, $c_{i}=150$)			0×B		
Epidemic?	1.00	0.99	0.08	1.00	1.00	0.00
Nb2. waves	0.00	0.02	0.16	0.00	0.03	0.18
Peak time	47.00	**65.77**	52.28	44.75	64.39	52.05
Peak size	2,873.28	*2,839.71*	1,509.91	2383.91	2,890.16	1,560.58
Time to curb*	47.00	68.27	53.39	46.00	67.52	53.10
Final size	1.00	**0.94**	0.12	0.99	0.92	0.13
	C ($a_{i}=20$, $d_{i}=50$)			0×C		
Epidemic?	1.00	1.00	0.03	1.00	1.00	0.00
Nb2. waves	0.00	0.13	0.35	0.00	0.13	0.33
Peak time	54.50	**71.83**	48.81	49.00	68.01	49.99
Peak size	2,757.37	*2,747.60*	1447.82	2386.43	2,849.68	1,468.74
Time to curb*	51.50	66.96	54.78	50.00	**69.96**	51.20
Final size	0.97	0.91	0.13	0.97	0.91	0.13
	D ($a_{i}=20$, $e_{i}=10$)			0×D		
Epidemic?	1.00	1.00	0.00	1.00	1.00	0.00
Nb2. waves	0.00	0.13	0.34	0.00	0.12	0.33
Peak time	39.75	59.80	40.92	49.00	**68.95**	52.40
Peak size	3,391.18	*3,126.29*	1,525.01	2,776.41	*2,805.00*	1,507.89
Time to curb*	39.50	59.28	43.90	50.50	**71.16**	53.62
Final size	1.00	**0.94**	0.10	0.97	0.91	0.13

† The statistics are based on n=3,840 simulations across the values of model parameters in Table 1.

* Time to curb the first epidemic wave Equation (11).

Italic (bold) figures indicate average values lower (larger) than the reference.

**TABLE 4:** Model fit results: variations of the number of secondary epidemic waves (Poisson model, log link), the peak size {gamma model, log link), and the final epidemic size (one-inflated beta model, logit link) across response profiles.

Response	Nb. secondary waves	Peak size	Final epidemic size
Term	Coefficient (SE)	Coefficient (SE)	Coefficient (SE)
(Intercept)	−16.055 (0.362)	7.449 (0.012)	5.210 (0.027)
Profile 0	0	0	0
Profile 0 × A	0.479 (0.068)	−0.046 (0.007)	−0.138 (0.015)
Profile A	0.734 (0.065)	−0.071 (0.007)	−0.196 (0.015)
Profile 0 × B	−1.013 (0.104)	−0.113 (0.007)	0.175 (0.016)
Profile B	−1.506 (0.127)	−0.132 (0.007)	0.673 (0.016)
Profile 0 × C	0.339 (0.070)	−0.102 (0.007)	−0.066 (0.015)
Profile 0 × C	0.399 (0.070)	−0.141 (0.007)	−0.080 (0.015)
Profile 0 × D	0.318 (0.071)	−0.133 (0.007)	−0.045 (0.015)
Profile D	0.159 (0.073)	−0.146 (0.007)	−0.038 (0.015)
$\alpha_{-1}$	−1.233 (0.031)	0.194 (0.002)	0.408 (0.004)
Δα	−0.217 (0.017)	0.063 (0.002)	0.074 (0.004)
κ	15.524 (0.362)	−0.541 (0.010)	−3.332 (0.021)
τ	−0.065 (0.007)	0.033 (0.001)	0.026 (0.002)
π	1.161 (0.092)	−0.771 (0.009)	−4.186 (0.021)
$\beta_{0}$	0.012 (0.017)ns	0.571 (0.002)	1.525 (0.004)
GOF: $\chi^{2}(df)$	7.011.37 (34,509)ns	4,772.52 (34,509)ns	2,8501.19 (34,507)ns
$R^{2}(\%)$ (%)	63.59	72.46	99.69

SE, standard error.

Profiles 0 and A to D are defined as follows: 0 is the reference (hence coefficient is fixed to 0) corresponding to a population responsive to prevalence only (i.e., $a_{i}=20$, with $b_{i}=c_{i}=d_{i}=e_{i}=0$ for i=−1,1), A corresponds to $a_{i}=20$, $b_{i}=50$, B corresponds to $a_{i}=20$, $c_{i}=50$, D corresponds to $a_{i}=20$, $b_{i}=50$, 0×A corresponds to half of the population is 0 and the other half is A; $\Delta \alpha =\alpha_{1}-\alpha_{-1}$, $\alpha_{-1}$, $\alpha_{1}$, κ, τ, π, and $\beta_{0}$ are model parameters defined in Table 1 with summary statistics given in Supplementary Table S1;ns indicates a non-significant test result at 5% level (i.e., the probability to observe an effect size equal to or bigger than the observed effect by random chance only is >5%); GOF, Goodness-of-fit; df, number of residual degrees of freedom; $\chi^{2}$ the deviance statistic which is expected to be at most of the order of df if the assumed model is appropriate; $R^{2}$ is the percentage of deviance from perfect fit explained by the included predictors as compared to no predictor. The dispersion parameter of the gamma distribution for peak size is 0.1074. For the final epidemic size, the dispersion parameter of the beta distribution is 0.1982, and the probability mass at one is 0.0045.
