# Supplementary material for: Heterogeneous risk tolerance, in-groups, and epidemic waves
Source: Front Appl Math Stat. Author manuscript; Available in PMC 2024 May 30. (PMC11138946; doi:10.3389/fams.2024.1360001)
Supplement: Supplementary Material [file NIHMS1992018-supplement-Supplementary_Material.pdf]

Supplementary Material

1 SUPPLEMENTARY TABLES AND FIGURES

1.1 Figures

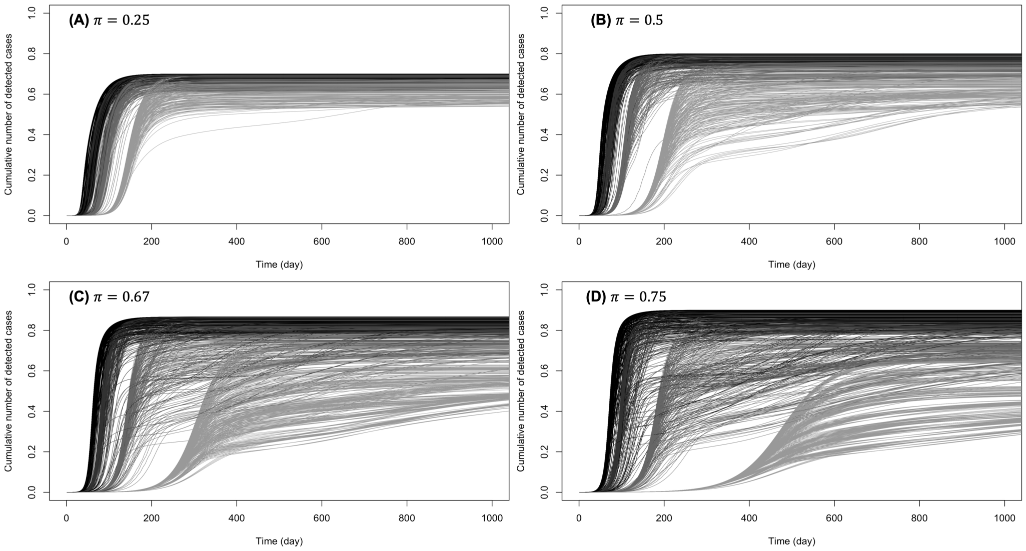

Figure S1: Cumulative number of detected cases when there is only one wave with (almost) perfect protection from prophylactic behavior: $\kappa \in \{0.95, 1\}$. For each probability of early detection of exposed individuals (π), the rightmost grey curves correspond to the lowest baseline disease transmission rate ($\beta_0 = 0.5$) and the dark curves (leftmost) correspond to the largest transmission rate ($\beta_0 = 3$). More or less grey curves have intermediate transmission rates ($\beta_0 = 1, 2$).

1.2 Tables

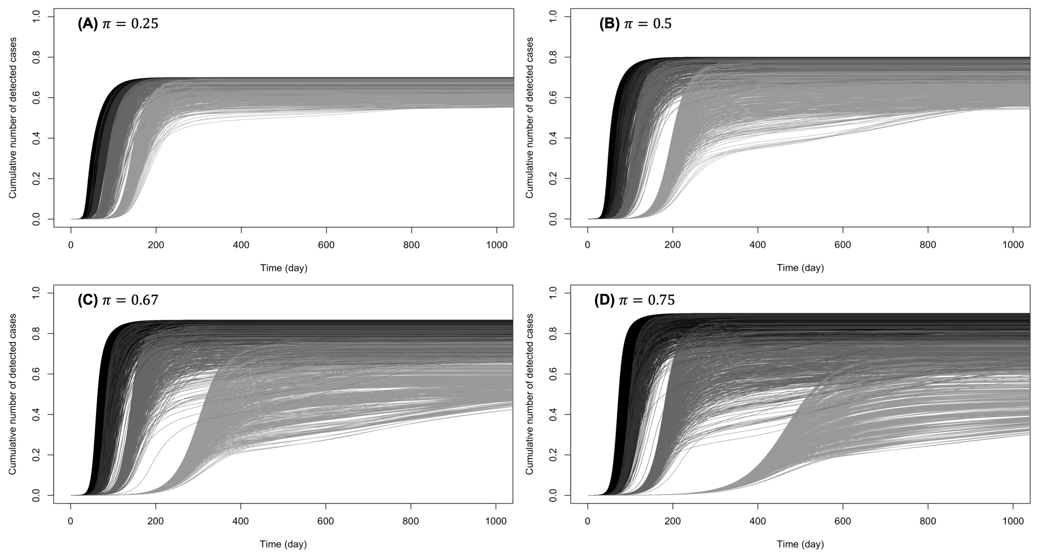

Figure S2: Cumulative number of detected cases when there is only one wave with high protection from prophylactic behavior: $\kappa \in \{0.75, 0.9\}$. For each probability of early detection of exposed individuals (π), the rightmost grey curves correspond to the lowest baseline disease transmission rate ($\beta_0 = 0.5$) and the dark curves (leftmost) correspond to the largest transmission rate ($\beta_0 = 3$). More or less grey curves have intermediate transmission rates ($\beta_0 = 1, 2$).

Table S1. Summary of numerical explanatory variables in the fitted models

**Statistics***	**Range**	**Inter-quartile range**	**Median**	**Mean**	**SD**
α_{-1}	[0.10, 3.00]	[0.10, 2.00]	1.00	1.04	0.96
$\Delta\alpha$	[0.00, 2.90]	[0.00, 1.90]	1.00	0.97	0.97
κ	[0.50, 1.00]	[0.60, 0.95]	0.75	0.78	0.18
τ	[1.00, 7.00]	[3.00, 7.00]	5.00	4.00	2.24
π	[0.25, 0.75]	[0.25, 0.67]	0.50	0.54	0.19
β_0	[0.50, 3.00]	[1.00, 3.00]	2.00	1.63	0.96

Table notes: SD = standard deviation; $\Delta\alpha = \alpha_1 - \alpha_{-1}$. Model parameters α_i, κ, τ, π, and β_0 are described in Table 1 of the main text. *The statistics are based on $n = 34560$ simulations across response profiles *0*, *A-D*.

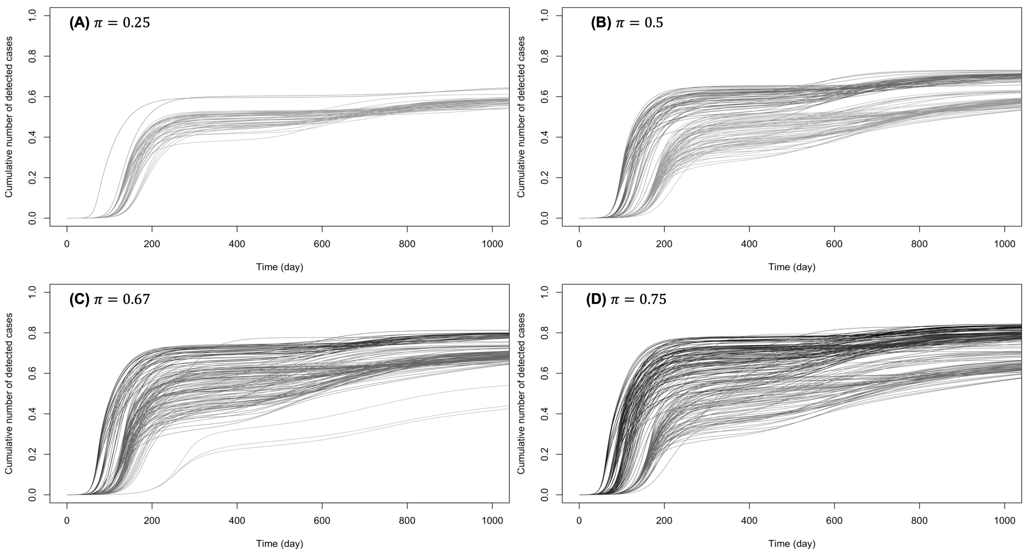

Figure S3: Cumulative number of detected cases when there are one or two secondary waves with high protection from prophylactic behavior: $\kappa \in \{0.75, 0.9\}$. For each probability of early detection of exposed individuals (π), the grey curves (rightmost) correspond to the lowest baseline transmission rate ($\beta_0 = 0.5$) and the dark curves (leftmost) correspond to the largest baseline transmission rate ($\beta_0 = 3$).

Table S2. Summary of epidemic severity measures across simulation scenarios

Statistics*	Range	Inter-quartile range	Median	Mean	SD
Epidemic?	[0.00, 1.00]	[1.00, 1.00]	1.00	1.00	0.03
Nb2. waves	[0.00, 1.00]	[0.00, 0.00]	0.00	0.11	0.31
T_p (days)	[19, 243]	[33, 81]	48	67	51
H_p (cases)	[147, 6415]	[1647, 4061]	2918	2920	1577
T_{c1} (days)	[1, 250]	[34, 83]	49	68	53
F_{1000} (%)	[43.8, 100.0]	[88.0, 100.0]	98.1	91.4	12.8

Table notes: SD = standard deviation; Nb2. waves = number of secondary waves; T_p = peak time, H_p peak height, T_{c_1} = time ti curb the first epidemic wave defined in equation (11) of the main text. *The statistics are based on $n = 34560$ simulations across response profiles *0*, *A-D*. Except for "Epidemic?", other statistics were computed given that "Epidemic?" = 1 (i.e. there is an epidemic outbreak).

Table S3. Summary of epidemic severity measures comparing populations with profiles *A*-*B* to the reference profile *0* under neutral in-group behavior ($\alpha_i = 1$)

**Statistics**†	**Median**	**Mean**	**SD**	**Median**	**Mean**	**SD**
	0 (Prevalence only, $a_i = 20$)					
Epidemic?	1.00	1.00	0.00			
Nb2. waves	0.00	0.11	0.32			
Peak time	42.00	62.46	50.28			
Peak size	3138.71	3150.07	1696.44			
Time to curb*	44.00	63.82	51.36			
Final size	0.97	0.90	0.14			
	A ($a_i = 20, b_i = 50$)			***0*** × ***A***		
Epidemic?	1.00	1.00	0.00	1.00	1.00	0.00
Nb2. waves	0.00	0.24	0.43	0.00	0.20	0.40
Peak time	41.50	60.88	48.16	41.50	61.56	49.03
Peak size	2960.01	2971.21	1696.62	3009.38	3049.77	1697.50
Time to curb*	42.50	62.15	49.15	43.00	62.98	50.09
Final size	0.96	0.90	0.14	0.97	0.90	0.14
	B ($a_i = 20, c_i = 150$)			***0*** × ***B***		
Epidemic?	1.00	0.99	0.10	1.00	1.00	0.00
Nb2. waves	0.00	0.02	0.18	0.00	0.02	0.15
Peak time	48.50	65.18	50.64	42.75	62.98	50.67
Peak size	2886.38	2767.71	1473.96	2807.49	2811.60	1526.49
Time to curb*	45.75	65.96	51.92	44.75	65.85	51.54
Final size	1.00	0.93	0.13	0.98	0.91	0.14
	C ($a_i = 20, d_i = 50$)			***0*** × ***C***		
Epidemic?	1.00	1.00	0.00	1.00	1.00	0.00
Nb2. waves	0.00	0.16	0.39	0.00	0.13	0.33
Peak time	57.25	71.90	46.92	47.75	66.51	48.57
Peak size	2485.84	2538.00	1306.69	2872.63	2792.55	1394.64
Time to curb*	51.00	63.93	54.39	48.00	68.02	49.56
Final size	0.96	0.90	0.13	0.96	0.90	0.13
	D ($a_i = 20, e_i = 10$)			***0*** × ***D***		
Epidemic?	1.00	1.00	0.00	1.00	1.00	0.00
Nb2. waves	0.00	0.10	0.30	0.00	0.12	0.32
Peak time	52.00	70.91	50.33	47.00	66.63	50.28
Peak size	2666.80	2613.61	1371.31	2790.43	2739.31	1412.19
Time to curb*	52.25	70.35	53.67	48.00	68.46	51.33
Final size	0.96	0.90	0.13	0.96	0.90	0.13

Table notes: SD = Standard Deviation; Nb2. waves = number of secondary waves. †The statistics are based on $n = 384$ simulations across the values of model parameters β_0, π, τ, and κ. *Time to curb = time to curb the first epidemic wave defined in (11) of the main text.
